# Surgical outcomes of single-port vs multi-port laparoscopic hysterectomy for endometrial cancer: A systematic review and meta-analysis

**DOI:** 10.1371/journal.pone.0314997

**Published:** 2024-12-09

**Authors:** Feifei Ji, Guansheng Chen, Mengyao Zhang, Xianying Chen, Jing Zhang, Dong Ding, Yongjun Wang

**Affiliations:** 1 Weifang Medical University, Weifang, China; 2 Beijing Jishuitan Hospital, Capital Medical University, Beijing, China; University of Bremen: Universitat Bremen, GERMANY

## Abstract

**Objective:**

This study aimed to compare the surgical outcomes in patients with endometrial cancer who underwent either single-port laparoscopic hysterectomy (SPLH) or multi-port laparoscopic hysterectomy (MPLH).

**Methods:**

We conducted a systematic literature search from the earliest records available up to May 2023. The databases searched included PubMed, Embase, ClinicalTrials.gov, and the Cochrane Library.

**Results:**

A total of 12 studies were included in the analysis. Both the SPLH and MPLH groups had similar operative times (MD = -4.27, 95% CI [-35.75, 27.22], p = 0.98), conversion rates (odds ratio [OR] = 1.43, 95% CI [0.57, 3.59], p = 0.44), blood transfusion rates, intraoperative complications (bladder injury, bowel injury, and vascular injury), and postoperative complications (umbilical hernia, fever, fistula, lymphocyst, and wound-related issues). However, the SPLH group showed significant advantages in certain areas. There was a notable reduction in estimated intraoperative blood loss (EBL) compared to the MPLH group (mean difference [MD] = -23.80, 95% CI [-42.99, -4.62], p = 0.02) and a shorter hospital stay duration (MD = -0.33, 95% CI [-0.46, -0.20], p < 0.00001). Although there was some debate about postoperative pain, SPLH tended to have more favorable outcomes. Despite these advantages, the SPLH group was less efficient in para-aortic lymph node clearance compared to the MPLH group (MD = -0.96, 95% CI [-1.57, -0.35], p = 0.002). No significant differences were observed in overall lymph node dissection (MD = -0.91, 95% CI [-2.52, 0.70], p = 0.27) and pelvic lymph node dissection (MD = -1.22, 95% CI [-3.82, 1.27], p = 0.36) between the two groups. Additionally, both groups showed similar therapeutic results, with no significant differences in overall survival (OS) and progression-free survival (PFS).

**Conclusion:**

SPLH and MPLH techniques are equally effective in treating endometrial cancer, with both showing low rates of surgical complications with similar rates of surgical complications and therapeutic outcomes. However, SPLH offers additional benefits, including smaller incisions, reduced estimated intraoperative blood loss, and shorter hospital stays, making it an increasingly popular option for treating endometrial cancer.

## Introduction

Endometrial cancer (EC), the most common gynecological malignancy in developed countries, is on the rise globally [1]. Although advanced-stage EC is associated with a poorer prognosis, early detection and diagnosis can significantly improve long-term patient outcomes [2]. Early diagnosis allows for curative surgery. The standard procedure for staging endometrial cancer is a total hysterectomy with bilateral salpingo-oophorectomy (TAH with BSO), sometimes combined with lymph node dissection and/or omental resection [3].

Several studies have shown that minimally invasive surgery (MIS) and open surgery have similar rates of progression-free survival (PFS) and overall survival (OS), indicating comparable long-term survival outcomes and risk of cancer recurrence [4–8]. However, MIS is generally associated with shorter surgical durations, reduced blood loss, and shorter hospital stays compared to open surgery [9]. The rise of laparoscopic techniques has led to the increasing adoption of MIS in managing EC [10]. Compared to open surgery, conventional laparoscopic surgery (CLS), also known as multi-port laparoscopy, offers more aesthetically pleasing incisions and a lower rate of incision infections [11]. However, CLS still results in three or distinct surgical scars. To improve the cosmetic benefits of minimally invasive surgery and reduce the potential morbidity from multiple incisions, laparoendoscopic single-port surgery (LESS) has been developed. LESS, though innovative, presents challenges such as instrument crowding, reduced depth perception, and the need for advanced laparoscopic skills [12]. Robotic single-port laparoscopic hysterectomy (RSPLH) addresses the depth perception issue but increases costs and preoperative preparation time. Randomized controlled trials (RCTs) for LESS and RSPLH in surgeries like cholecystectomy and appendectomy show that while these techniques offer cosmetic and pain benefits, their clinical outcomes are often similar to traditional laparoscopy [13, 14]. The question of whether SPLH offers superior surgical outcomes compared to MPLH for endometrial cancer remains unresolved.

Our systematic review and meta-analysis aim to bring together the large amount of research on SPLH and MPLH for treating EC. We want to carefully look at and explain any differences in surgical outcomes between these two methods. The goal of our research is to offer valuable insights that can help with clinical decision-making and ultimately improve the well-being of EC patients.

## Methods

### Literature search strategy

Authors Ji and Chen meticulously conducted a meticulous and comprehensive literature search, adhering to the 2020 Preferred Reporting Items for Systematic Reviews and Meta-Analyses (PRISMA) Checklist [15], as presented in S2 Table. The meta-analysis and systematic review are registered on the PROSPERO website under registration number CRD42024516924. The search encompassed renowned databases, including PubMed, Embase, ClinicalTrials.gov, and the Cochrane Library, covering the period from their inception until May 2023. Search terms included synonyms and abbreviations related to endometrial cancer, single-port laparoscopy, and hysterectomy. Details of the search strategy are presented in S1 Table.

### Inclusion and exclusion criteria

The inclusion criteria required the selected studies to meet the following conditions:

1. Patients were diagnosed with endometrial cancer;
2. Observational studies or randomized controlled trials;
3. One group was treated with single-port laparoscopy, including traditional single-port laparoscopy, robotic single-port laparoscopy, or both. The control group was treated with multi-port laparoscopy, including traditional multi-port laparoscopy, robotic multi-port laparoscopy, or both, with comparisons made between the two groups.

Excluded studies included:

1. Hysterectomy performed only for benign conditions;
2. Articles comprising case reports, reviews, meta-analyses, organizational guidelines, letters, expert opinions, or meeting abstracts;
3. Studies that lacked sufficient data that could be extracted or calculated.

### Data extraction

Authors Ji and Chen independently extracted data from all eligible articles using standardized methods. Any discrepancies between them were resolved through discussion and, if necessary, reviewed by a third independent reviewer. The extracted information included key details such as the first author’s name, publication year, baseline patient characteristics, study design, participant numbers in both the experimental and control groups, and experimental outcomes. The gathered data was meticulously entered into the Review Manager software for subsequent analysis [16].

### Quality assessment

In this meta-analysis, we employed the Newcastle-Ottawa Scale to assess the quality of non-randomized controlled studies [17]. This assessment covers three primary dimensions and nine scoring items: study population, comparability between groups, and outcome measurement. Within these dimensions, the study population includes four scoring items, comparability between groups encompasses two, and outcome measurement comprises three. Each article receives a total quality assessment score out of 9 points, with scores ranging from 0 to 4 indicating low-quality literature and scores from 5 to 9 representing high-quality literature. To assess the risk of bias in the two randomized controlled trials, we used the modified Jadad score [18]. This evaluation was based on four criteria: the generation of random sequences, concealment of allocation, blinding, and withdrawals/dropouts. Studies scoring 4 or higher were considered high quality.

Independent assessments of study quality were conducted by two authors who cross-verified their evaluations. Additionally, we assessed potential biases within these studies. For assessing the quality of the study outcomes, we utilized TSA (Trial Sequential Analysis) and GRADE (Grading of Recommendations Assessment, Development and Evaluation) grading to evaluate the quality of the literature included in our review [19, 20].

### Statistical analysis

Meta-analyses were conducted using Review Manager version 5.4 (Cochrane Collaboration, Oxford, UK). For dichotomous comorbidity outcomes, odds ratios (OR) and their corresponding 95% confidence intervals (CI) were calculated. Statistical heterogeneity, which reflects variability in intervention effects across trials, may arise from clinical or methodological variations or both. Heterogeneity was assessed using the Chi-square test and the I^2^ test [21, 22]. When significant heterogeneity was detected (p-value < 0.05 or I^2^ > 50%), a random-effects model was employed to calculate pooled effects. Conversely, if no significant heterogeneity was identified, a fixed-effects model was used.

## Results

### Search results

**Fig 1:**
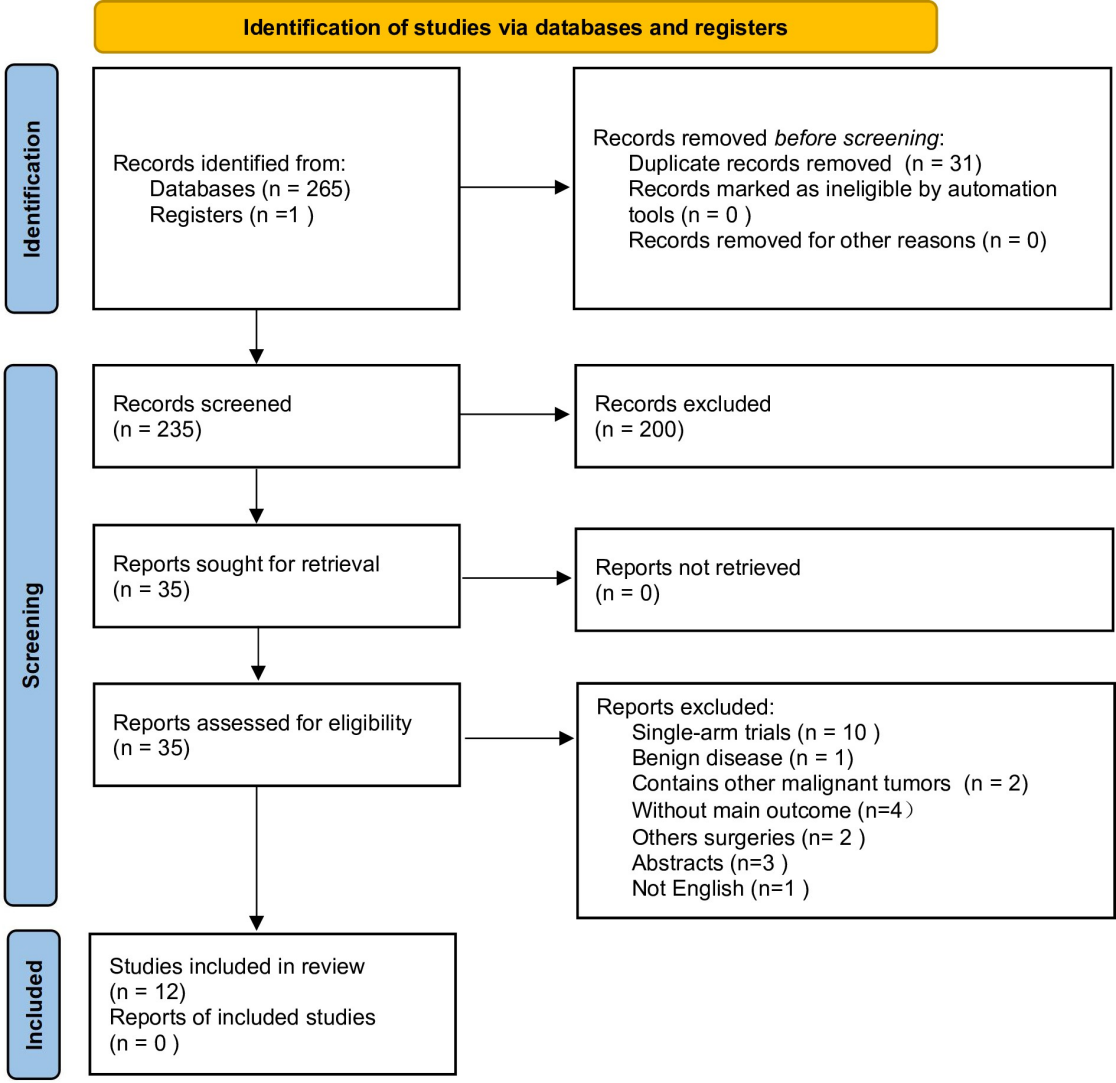
PRISMA flow diagram.

Fig 1 presents a flowchart illustrating the literature screening process. Initially, a total of 266 studies were retrieved from PubMed, Embase, ClinicalTrials.gov, and the Cochrane Central Register of Controlled Trials (CENTRAL). These studies were imported into ENDNOTE X9 for processing and screening, resulting in the removal of 31 duplicate studies. The remaining articles were then evaluated based on their titles and abstracts to determine their compliance with the selection criteria. Consequently, 35 articles were selected for a more comprehensive assessment by reviewing their full text. After this rigorous review, 12 studies met the inclusion criteria and were included in the final analysis.

### Description of studies

Table 1 provides an overview of the characteristics of the included studies, detailing the country of origin, publication year, study design, and procedure type. Among the included studies, 2 studies were RCTs [11, 23], 3 studies were prospective studies [11, 24–26], and 7 studies were retrospective studies [27–33]. These studies collectively involved 463 women who underwent SPLH and 651 women who underwent MPLH. In total, the analysis included comparisons from 12 studies: 7 studies compared LESS to MPL, 4 studies compared RSPLH to RMPLH, and 1 study compared LESS to RMPLH. Tables 2 and 3 present the quality assessments of the included non-randomized controlled studies and randomized studies, respectively.

**Table 1:** Characteristics of the included studies.

Author	Year	Country	Study type	No. of centers	Procedure and patients		Patient population
Cai et al.[23]	2021	China	RCT^a^	1	LESS^b^: 31	CMPLH^c^:62	FIGO stage Ⅰ
Kang et al. [11]	2023	Korea	RCT	1	LESS: 53	CMPLH: 54	FIGO stage IA, IB
Dou et al. [24]	2021	China	Retrospective	1	LESS: 48	CMPLH: 58	FIGO stage IA, IB,Ⅱ
Escobar et al. [25]	2011	American	Retrospective	3	LESS: 30	CMPLH:30	FIGO stage IA, IB,IC,Ⅱ
Corrado et al. [26]	2016	Italy	Retrospective	1	RSPLH^d^: 23	RMPLH^e^:46	FIGO stage IA, IB
Corrado et al. [27]	2020	Italy	Retrospective	4	RSPLH:71	RMPLH:149	FIGO stages IA,ⅠB, II
Moukarzel et al. [28]	2017	USA	Retrospective	1	RSPLH: 14	RMPLH: 13	CAH*, FIGO Stage IA, IB
Fagotti et al. [29]	2012	Italy	Retrospective	4	LESS: 75	RMPLH: 75	FIGO stage IA,IB, II, IIIA ,IIIC
Cai et al. [30]	2016	China	Retrospective	1	LESS: 18	CMPLH:18	FIGO stage IA
Park et al. [31]	2014	Korea	Prospective	1	LESS: 37	RMPLH: 74	FIGO stage IA,IB, IIA, IIIC1 ,IIIC2
Mereul et al. [32]	2022	Italy	Prospective	3	RSPLH:25	RMPLH: 51	FIGO stage IA,IB,IIIB ,IIIC
Gloria et al. [33]	2017	Italy	Perspective	1	RSPLH:15	RMPLH: 13	FIGO stage IA,IB

Note: a RCT: Randomized Controlled Trial, b LESS: Laparoscopic-Endoscopic Single-Site Surgery, C CMPLH: Conventional multi-port laparoscopic hysterectomy, d RSPLH: Robotic single-port laparoscopic hysterectomy, e RMPLH: Robotic multi-port laparoscopic hysterectomy.

**Table 2:** Quality assessment of the included Non-RCT studies according to NOS score.

Author	Year	Selection	Comparability	Outcome	Quality score
Dou et al.	2021	***	**	***	8
Escobar et al.	2011	****	**	**	8
Corrado et al.	2016	****	**	***	9
Corrado et al.	2020	****	**	*	7
Moukarzel et al.	2017	****	**	***	9
Fagotti et al.	2012	***	**	**	7
Cai et al.	2016	****	**	***	9
Park et al.	2014	***	**	***	8
Mereul et al.	2022	***	**	**	7
Gloria et al.	2017	***	**	***	7

**Table 3:** Quality assessment of the included RCT studies according to modified Jadad score.

Study	Was the study described as randomized?	Was the method of randomization appropriate?	Was the study described as blinded?	Was the method of blinding appropriate?	Was there a description of withdrawals and dropouts?	Was there a clear description of the inclusion/exclusion criteria?	Was the method used to assess adverse effects described?	Was the method of statistical analysis described?	Modified Jadad Score
Cai et al.	1	1	0	0	1	1	1	1	6
Kang et al.	1	1	1	1	1	1	1	1	8

### Operative time

**Fig 2:**
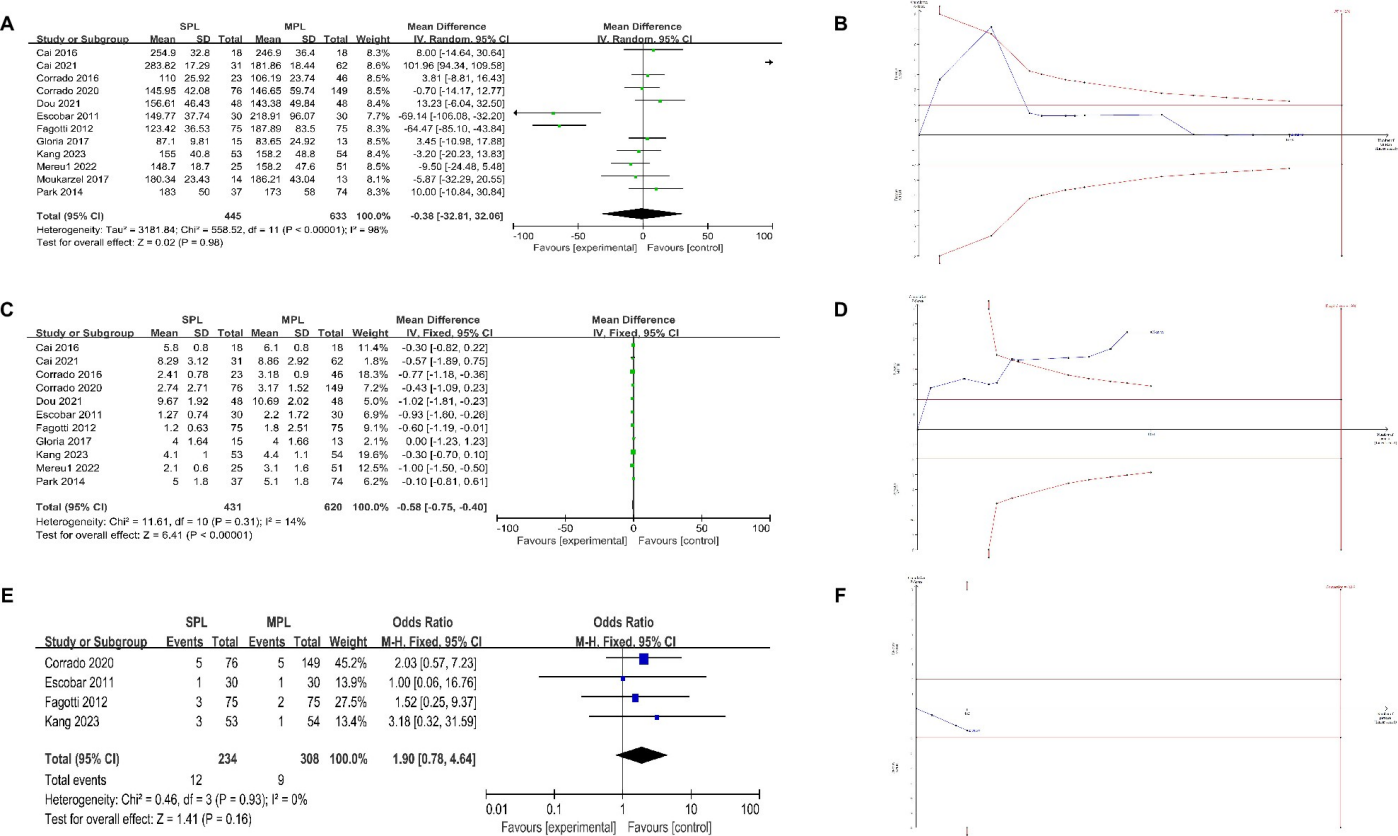
Meta-analysis results of operative time, hospital stay, conversion, and trial sequential analysis (TSA) of this results. (A) operative time, (B) TSA of operative time, (C) hospital stay, (D) TSA of hospital stay, (E) conversion, (F) TSA of conversion. Note: In TSA, the alpha spending function is automatically calculated after adjusting for Type I and Type II errors, and the statistical monitoring boundaries are shown in the TSA plot. There are four types of lines in TSA analysis:
The blue line (Z-curve) represents the TSA analysis results, with the last point determining the meta-analysis outcome.The horizontal dark red line represents the conventional threshold. If the last point of the Z-curve falls between the lines, it indicates no significant difference between the groups in the meta-analysis.The red diagonal line represents the TSA trial boundary. If the last point of the Z-curve crosses the conventional boundary but not the trial boundary, the meta-analysis is significant, but the TSA analysis is not. If the last point crosses the trial boundary, both the meta-analysis and TSA analysis are significant.The final vertical red line represents the required information size (RIS) for the current conclusion. The closer the sample size in the meta-analysis and TSA analysis is to the RIS, the stronger the evidence; the further away, the weaker the evidence.

Fig 2A presents the results regarding operative time. The mean operative time showed no significant difference between the SPL group and the MPL group (MD = -0.38, 95% CI [-32.81, 32.06], p = 0.98). It is noteworthy that significant heterogeneity was observed among the included studies (I^2^ = 98%, p < 0.00001). The corresponding information in Table 4 demonstrates that subgroup comparisons of LESS vs. MPLH (MD = 11.17, p = 0.68) and RSPLH vs. RMPLH (MD = -0.63, p = 0.85) did not yield significant differences. After adjusting for type 1 and type 2 errors in the TSA, differences in operative time between the two groups may exist. Graphically observed in Fig 2B, the z-curve (blue line) intersects both the traditional threshold (horizontal deep red line) and the trial sequence boundary (red diagonal line). TSA also indicates that the power of such findings is insufficient due to the analyzed number of patients for surgical time (1078), which is lower than the calculated 1230 patients needed currently.

**Table 4:** Subgroup analysis of surgical methods.

Comparison	Types	No. of studies	OR/MD	95% CI	Overall effect P-value	I^2^ %	Heterogeneity p-value
Operation time	LESS vs CMPLH	6	11.17	-41.80, 64.14	0.68	98	<0.00001
	RSPLH vs RMPLH	5	-0.63	-7.30, 6.04	0.85	0	0.68
Estimated blood loss	LESS vs CMPLH	6	-0.11	-0.42, 0.20	0.49	65	0.01
	RSPLH vs RMPLH	4	-0.52	-0.86, -0.18	0.003	39	0.18
Postoperative complications	LESS vs CMPLH	6	0.87	0.50, 1.53	0.63	31	0.23
	RSPLH vs RMPLH	3	0.73	0.29, 1.83	0.50	0	0.74
Transfusion	LESS vs CMPLH	3	0.87	0.25, 3.08	0.83	0	0.69
	RSPLH vs RMPLH	2	0.38	0.04, 3.33	0.38	0	0.99
Hospital stay	LESS vs CMPLH	6	-0.45	-0.69, -0.20	0.0004	17	0.30
	RSPLH vs RMPLH	4	-0.63	-0.96, -0.29	0.0002	5	0.37

### Length of hospital stay

Fig 2C illustrates the results regarding the length of hospital stay. Compared to the MPLH group, the SPLH group showed a significantly shorter duration of hospitalization (MD = -0.58, 95% CI [-0.75, -0.40], p < 0.00001), with minimal heterogeneity across studies (I^2^ = 14%, p = 0.31). Further details from subgroup analysis (Table 4) highlight that LESS demonstrated a shorter hospital stay compared to CMPL (MD = -0.45, p = 0.0004), while RSPLH also demonstrated a shorter hospital stay compared to RMPLH (MD = -0.63, p = 0.0002). These results were also confirmed in the TSA analysis (Fig 2D), where, after adjusting for type 1 and type 2 errors, the z-curve intersects both the traditional threshold (horizontal deep red line) and the trial sequence boundary (red diagonal line). However, due to the lower number of analyzed hospital stay patients (1051) compared to the expected sample size (1906), the TSA analysis indicates limited power for these findings.

### Conversion

Fig 2E presents the findings on the conversion rate, indicating no discernible difference between the SPL group and the MPL group (OR = 1.43, 95% CI [0.57, 3.59], p = 0.44). Notably, no substantial heterogeneity was observed among the studies (I^2^ = 0%, p = 0.77). These results were confirmed after adjusting for type 1 and type 2 errors in the TSA. In Fig 2F, the lack of statistical significance is illustrated, as final value of the z-curve (blue line) is below both the conventional threshold (horizontal deep red line) and the trial sequence boundary (red diagonal line). These findings are characterized by very low evidence strength, as the included number of patients (482) is lower than the calculated 4016 patients.

#### Estimated blood loss and transfusion

**Fig 3:**
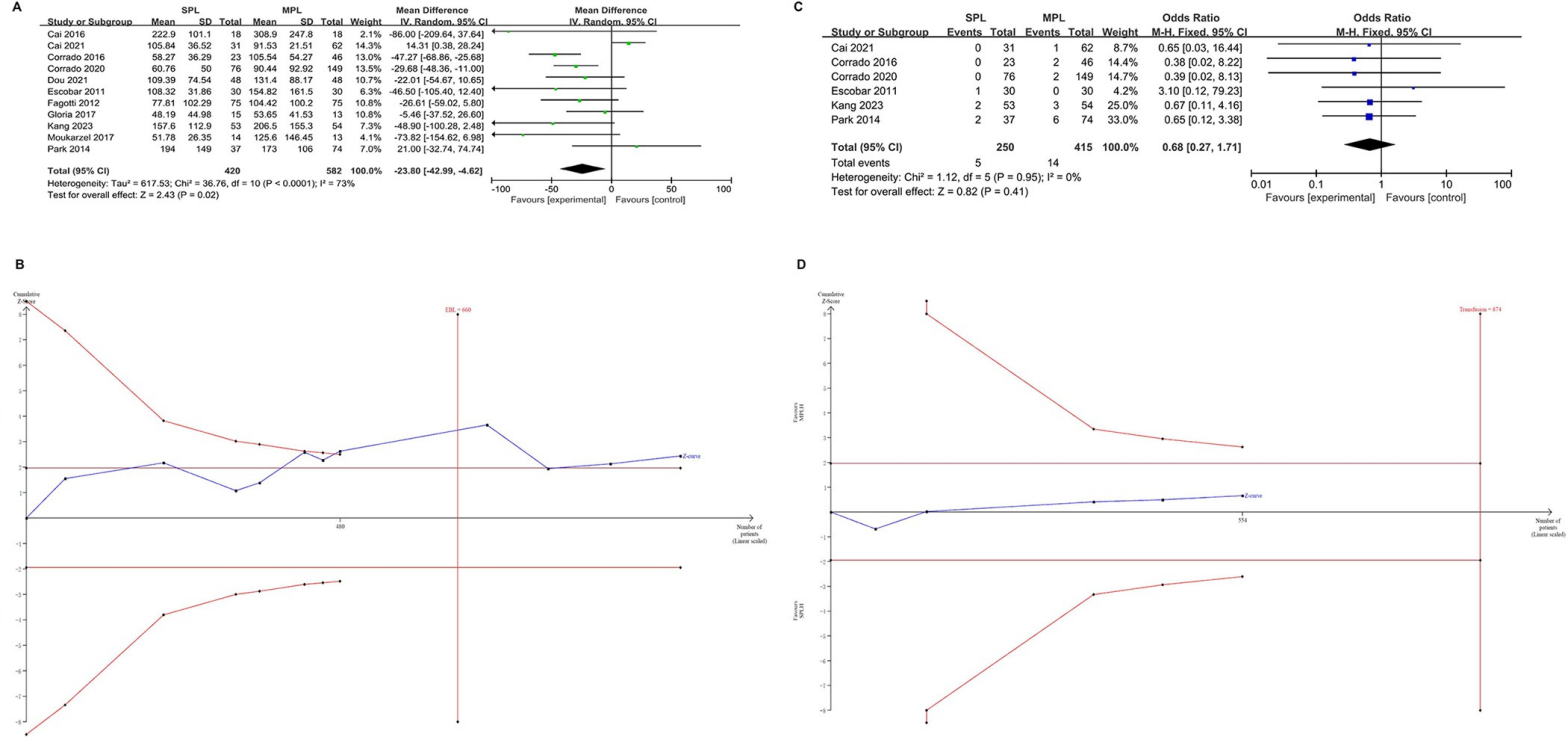
Meta-analysis results of estimated blood loss (EBL), transfusion, and trial sequential analysis (TSA) of this results. (A) EBL, (B) TSA of EBL, (C) transfusion, (D) TSA of transfusion.

Fig 3A presents the results regarding estimated blood loss (EBL). A significantly lower EBL was observed in the SPLH group compared to the MPLH group (MD = -23.80, 95% CI [-42.99, -4.62], p = 0.02), with a high level of heterogeneity among the studies (I^2^ = 73%, p < 0.0001). These results were confirmed after adjusting for type 1 and type 2 errors in the TSA. The lack of statistical significance in the TSA can also be observed graphically in Fig 3B, where the z-curve (blue line) intersects both the conventional threshold (horizontal deep red line) and the trial sequence boundary (red diagonal line). TSA indicates that the power of these findings is insufficient due to the lower number of patients with estimated blood loss (480) compared to the required 660 patients. Detailed findings from the subgroup analysis (see Table 5) revealed no significant difference in EBL between LESS and MPL (MD = -15.55, p = 0.29). However, RSPLH exhibited a significant reduction in EBL compared to RMPLH (MD = -32.52, p = 0.0001). The odds ratio (OR) for blood transfusion did not show statistical significance among the groups (OR = 0.68, 95% CI [0.23, 1.99], p = 0.48) (Fig 3C), and homogeneity characterized the studies (I^2^ = 0%, p = 0.89). After adjusting for type 1 and type 2 errors in the TSA, these results were also confirmed. In Fig 3D, the lack of statistical significance is graphically shown because the final value of the z-curve (blue line) is below both the conventional threshold (horizontal deep red line) and the trial sequence boundary (red diagonal line). These findings are characterized by very low evidence strength, as the included number of transfusion patients (554) is lower than the calculated 874.

**Table 5:** Outcomes of postoperative pain.

Authors	Approach	Single-port	Multi-port	Outcomes	Significant
Park et al. 2014	LESS vs CMPLH	37	74	16H, 24H, 32H, LESS favored	Yes
Cai et al. 2021	LESS vs CMPLH	31	62	24H, LESS favored	Yes
Kang et al. 2023	LESS vs CMPLH	53	54	Comparable	NO
Dou et al. 2021	LESS vs CMPLH	48	48	Comparable	NO
Gloria 2018	RSPLH VS RMPLH	15	13	Comparable	NO
Mereu1 et al. 2022	RSPLH VS RMPLH	25	51	12H , RMPLH favored	Yes

### Postoperative pain

Table 5 shows all postoperative pain outcomes. Most studies suggest that SPLH and MPLH have comparable outcomes in terms of postoperative pain. However, studies by Parker et al. [31] and Cai et al. [23] indicate a trend of reduced postoperative pain with LESS. Conversely, Meruzul et al. [32] found lower postoperative pain scores with MPLH. Based on these research findings, patients with SPLH may exhibit lower levels of postoperative pain. However, further prospective randomized controlled studies are needed to confirm these results.

#### Lymph node dissection

**Fig 4:**
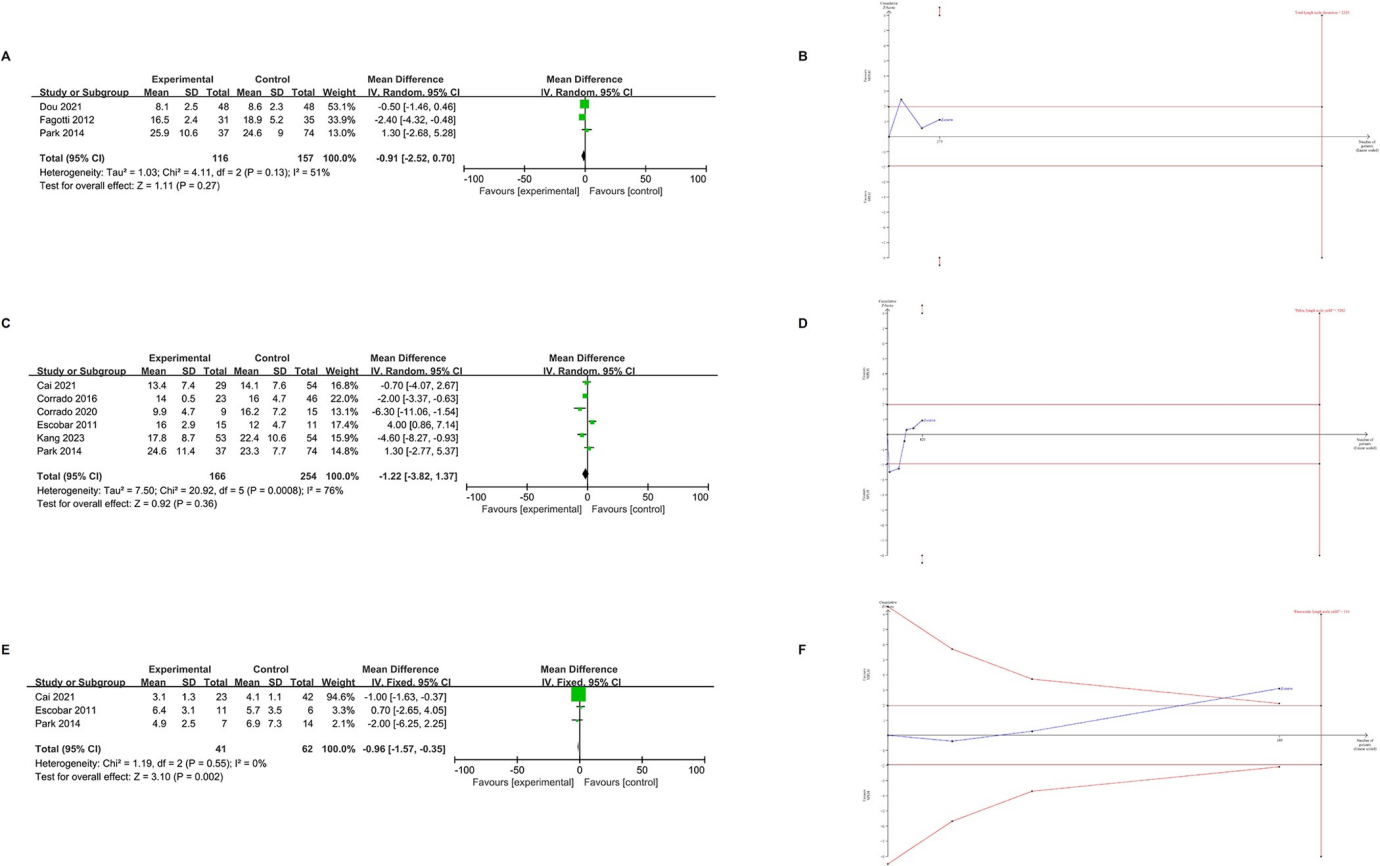
Meta-analysis results of lymph node dissection, and trial sequential analysis (TSA) of this results. (A) total intraoperative lymph nodes dissection, (B) TSA of total intraoperative lymph nodes dissection, (C) pelvic lymph nodes dissection, (D) TSA of pelvic lymph nodes dissection, (E) para-abdominal aortic lymph node dissection, (F) TSA of para-abdominal aortic lymph node dissection.

Fig 4A, 4C, and 4E present the results comparing SPLH to MPLH regarding total lymph node dissection (MD = -0.91, 95% CI [-2.52, 0.70], p = 0.27), pelvic lymph node dissection (MD = -1.22, 95% CI [-3.82, 1.27], p = 0.36), and para-aortic lymph node dissection (MD = -0.96, 95% CI [-1.57,-0.35], p = 0.002). Both procedures show comparable results for overall and pelvic lymph node dissection efficiency. The TSA for both total lymph node dissection and pelvic lymph node dissection also supports the above results (Fig 4B and 4D). After adjusting for type 1 and type 2 errors, the blue line of the z-curve crossed the traditional threshold (horizontal deep red line) but did not cross the trial sequence boundary (red diagonal line). However, the evidence strength is very low because the number of patients undergoing total lymph node dissection (273) and pelvic lymph node dissection (420) is lower than the calculated required samples of 2329 and 5200, respectively. MPLH demonstrates superior efficiency in para-aortic lymph node dissection. The improved efficiency in para-aortic lymph node dissection does not seem to have a significant impact on the late-stage prognosis of patients. For para-aortic lymph node dissection, the TSA results were confirmed after adjusting for type 1 and type 2 errors. The blue line of the z-curve simultaneously intersects both the conventional threshold (horizontal deep red line) and the trial sequence boundary (red diagonal line). The TSA also indicates that the power of such findings is insufficient, as the number of patients undergoing para-aortic lymph node dissection (103) is lower than the calculated 114 patients needed.

### Complications

**Fig 5:**
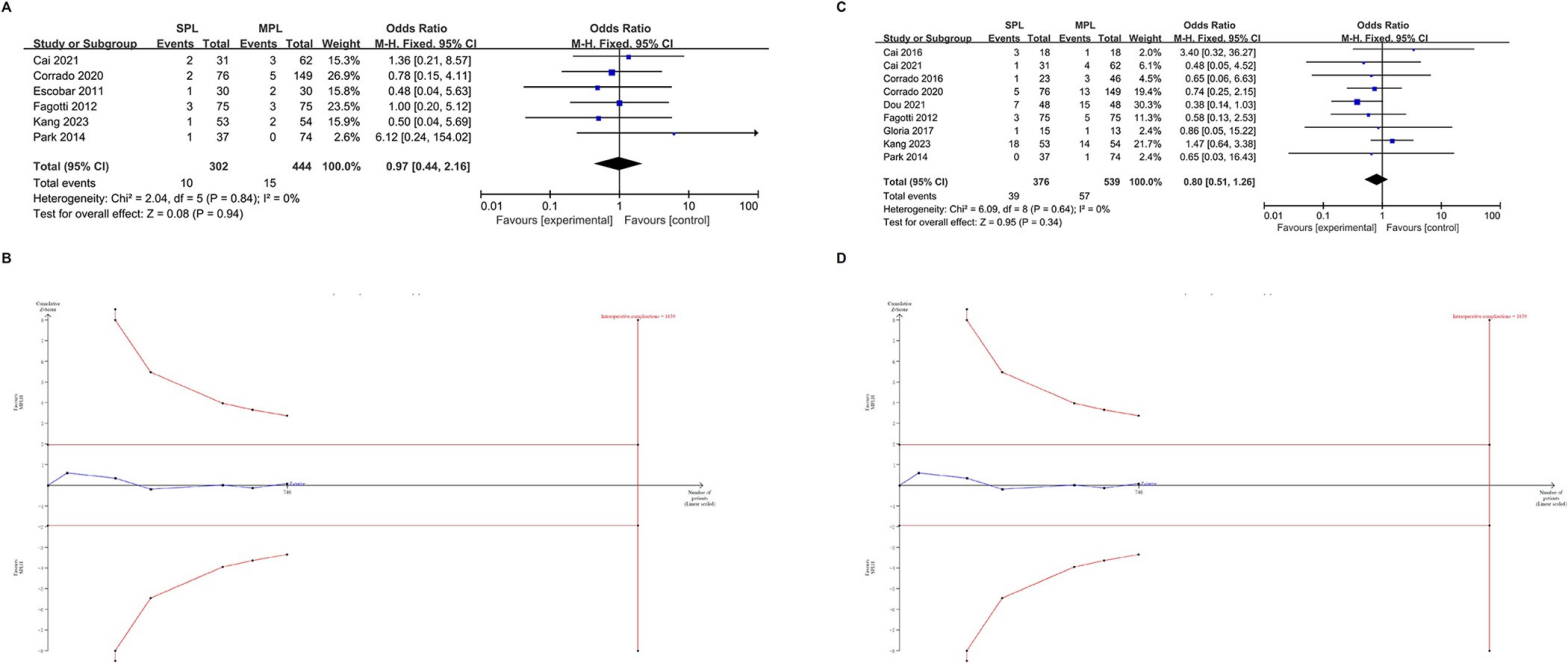
Meta-analysis results of complications, and trial sequential analysis of complications. (A) intraoperative complications, (B) TSA of intraoperative complications, (C) postoperative complications, (D) TSA of postoperative complications.

Fig 5A presents the outcomes related to intraoperative complications. The overall rates of intraoperative complications showed no significant difference between the SPLH group and the MPLH group (OR = 0.97, 95% CI [0.42, 1.95], p = 0.94), with no heterogeneity observed across the studies (I^2^ = 0%, p = 0.87). Table 6 provides further insights into individual intraoperative complications, revealing no significant differences in bladder injury (OR = 0.84, p = 0.90), bowel injury (OR = 0.45, p = 0.50), and vascular injury (OR = 1.40, p = 0.64) between the SPLH and MPLH groups. In Fig 5B, the final value of the z-curve (blue line) is below both the conventional threshold (horizontal deep red line) and the trial sequence boundary (red diagonal line). The evidence strength is very low because the number of transfusion patients included (746) is lower than the calculated 1839.

**Table 6:** Complications subgroup analysis results.

Comparison	No. of studies	OR	95% CI	Overall effect P-value	I^2^ %	Heterogeneity p-value
Bladder injury	4	1.11	0.22, 5.51	0.90	0	0.42
Bowel injury	2	0.45	0.05, 4.44	0.50	0	0.76
Vascular injury	4	1.35	0.36, 5.06	0.65	0	0.58
Would problem	4	0.68	0.27, 1.73	0.42	0	0.75
Fever	4	1.45	0.62, 3.39	0.39	0	0.97
Lymphocele	2	0.37	0.06, 2.22	0.28	0	0.92
Fistula related complications	2	0.48	0.05, 4.39	0.52	0	0.82

In terms of postoperative complications, as depicted in Fig 5B, no statistically significant differences were observed between the two groups (OR = 0.82, 95% CI [0.54, 1.24], p = 0.59). Subgroup analyses, as outlined in Table 4, revealed no statistically significant differences between subgroups when comparing LESS with CMPL (OR = 0.85, p = 0.54) or RSPLH with RMPLH (OR = 0.72, p = 0.47). Additionally, detailed assessment of individual postoperative complications, as presented in Table 6, including umbilical hernia (OR = 0.29, p = 0.27), fever (OR = 1.50, p = 0.37), fistula (OR = 0.48, p = 0.52), lymphocyst (OR = 0.30, p = 0.27), and wound-related issues (OR = 0.64, p = 0.33), showed no substantial differences between the two groups. In Fig 5D, the final value of the z-curve (blue line) is below both the conventional threshold (horizontal deep red line) and the trial sequence boundary (red diagonal line). However, the TSA indicates that the power of these findings is sufficient because the number of postoperative inpatient patients included in the study is higher than the calculated 409 patients needed.

### Long-term outcomes

As shown in Table 7, four studies reported the postoperative overall survival (OS) for SPLH and MPLH, and three studies reporting the progression-free survival (PFS) for both. The results indicate comparable outcomes for both SPLH and MPLH in terms of postoperative OS and PFS, with no statistically significant differences.

**Table 7:** Long-term outcomes.

Author	Approach	Single port	Multi-port	Outcomes	Significant
OS					
Corrado et al. 2020	LESS vs CMPLH	37	74	Comparable	NO
Cai et al. 2021	LESS vs CMPLH	31	62	Comparable	NO
Kang et al.2023	LESS vs CMPLH	53	54	Comparable	NO
Dou er al. 2021	LESS vs CMPLH	48	48	Comparable	NO
PFS					
Cai et al.2021	LESS vs CMPLH	31	62	Comparable	NO
Kang et al. 2023	LESS vs CMPLH	53	54	Comparable	NO
Dou et al. 2021	LESS vs CMPLH	48	48	Comparable	NO

### Quality of the evidence

The evidence grading for SPLH in the treatment of endometrial cancer is shown in Table 8. The results indicate that the quality of evidence for hospital stay, estimated intraoperative blood loss, transfusion rate, total lymph node dissection, intraoperative complications, and postoperative complications. However, the quality of evidence for operative time, conversion rate, nausea, and vomiting is rated as very low.

**Table 8:** Evaluation of evidence using GRADE criteria.

Outcomes	NO. of studies	Certainty assessment					No. of patients		Certaintyty
		Risk of bias	Inconsistency	Indirectness	Imprecision	Other considerations	SPLH	MPLH	
Operative time	2 RCTs+ 10 Non RCTs	serious ^a^	serious ^b^	not serious	not serious	not serious	420	582	⨁◯◯◯ Very low
Hospital stay	2 RCTs+ 9 Non RCTs	serious^ a^	not serious	not serious	not serious	not serious	406	569	⨁⨁◯◯ Low
Estimated Blood Loss	2 RCTs+ 9 Non RCTs	serious ^a^	not serious	not serious	not serious	not serious	420	582	⨁⨁◯◯ Low
Conversion	1 RCT+ 3 Non RCTs	serious ^a^	not serious	not serious	serious^c^	not serious	234	308	⨁◯◯◯ Very low
Transfusion	2 RCTs+ 4 Non RCTs	serious^ a^	not serious	not serious	not serious	not serious	250	415	⨁⨁◯◯ Low
Nausea and vomiting	2 RCTs+ 1 Non RCT	serious ^a^	not serious	not serious	serious ^c^	not serious	86	85	⨁◯◯◯ Very low
Pelvic lymph node dissection	2 RCTs+ 4 Non RCTs	serious ^a^	not serious	not serious	not serious	not serious	166	254	⨁⨁◯◯ Low
Intraoperative complications	2 RCTs+ 4 Non RCTs	serious ^a^	not serious	not serious	not serious	not serious	302	444	⨁⨁◯◯ Low
postoperative complications	2 RCTs+ 7 Non RCTs	serious ^a^	not serious	not serious	not serious	not serious	376	539	⨁⨁◯◯ Low

Note: GRADE Working Group grades of evidence: High quality: Further research is very unlikely to change our confidence in the estimate of effect. Moderate quality: Further research is likely to have an important impact on our confidence in the estimate of effect and may change the estimate. Low quality: Further research is very likely to have an important impact on our confidence in the estimate of effect and is likely to change the estimate. Very low quality: We are very uncertain about the estimate.

a The GRADE level was changed as follows: Certainty in the evidence downgraded by one level due to serious inconsistency; certainty in the evidence downgraded by two levels due to very serious inconsistency; and certainty in the evidence downgraded by one level due to serious imprecision. The inconsistency was defined by the high value of I^2^. The imprecision was defined by confidence interval.

b Based on the authors reporting no publication bias.

c The number of studies were insufficient to preform analysis.

## Discussion

Endometrial cancer is the most common malignancy of the female reproductive tract in developed countries. Most patients are diagnosed after experiencing abnormal postmenopausal bleeding, and 5% of them have not yet had children [34]. For women who wish to conceive in the future, conservative treatment should be considered first. Initially, fertility-sparing treatment was mainly used for patients with low-grade, stage 1 endometrial cancer [35]. However, a recent article by Andrea Etrusco et al. [36] suggests that fertility-sparing treatment may also be a safe and feasible option for women of childbearing age with G2 endometrial cancer. For postmenopausal patients and those with high-grade cancer, aggressive interventions are necessary. Surgical intervention remains the primary treatment for endometrial cancer. In recent years, advancements in laparoscopic techniques and the development of surgical instruments have highlighted their benefits, such as less trauma, decreased intraoperative bleeding, and expedited postoperative recovery [37]. Notably, laparoscopic surgery provides comparable outcomes to open surgery in terms of cancer recurrence and long-term survival [38].

A recent innovation in minimally invasive surgery is transumbilical single-port laparoscopic surgery (SPLS) procedure. SPLS offers advantages over conventional laparoscopy in gynecology, including less post-operative pain, faster recovery, better cosmetic results, and shorter hospital stays [31]. However, SPLS has its challenges, such as the "chopstick effect,” where all instruments are inserted through single incision [39]. This can lead to interference between the instruments and the optical system, resulting in unstable and unclear visualization. The coaxial manipulation required in SPLS may also reduce the precision of surgical maneuvers, especially when assessing depth during procedures [40].

Improvements in instrument design have enhanced precision and maneuverability. The use of ultrasonic cutting and coagulating devices, like the Harmonic scalpel, allows for more efficient tissue dissection and hemostasis, reducing operative time and blood loss [41]. Additionally, the availability of instruments with different handle lengths helps address the anatomical challenges of endometrial cancer, providing surgeons with more flexibility [42]. Smaller and lighter endoscopic cameras have also improved visualization, making SPLS safer and more minimally invasive [43].

The effectiveness and safety of SPLH for benign gynecological tumors are well-established, and its cosmetic benefits have garnered interest. However, debates continue regarding its superiority over multi-port laparoscopy for endometrial cancer due to factors like surgical complexity, equipment cost, surgeon expertise, and ethical considerations [44]. There is limited literature comparing single-port and multi-port techniques specifically for treating gynecological malignancies like endometrial cancer.

Our meta-analysis found that the single-port group had significantly lower estimated intraoperative blood loss compared to the multiport group. To provide a comprehensive assessment, we included studies regardless of robot assistance and conducted subgroup analyses. These analyses showed that LESS did not significantly reduce intraoperative blood loss, while RSPLH did, consistent with previous studies. The reduced intraoperative bleeding observed in RSPLH may be due to less local trauma, increased precision in surgical maneuvers, and smaller incisions.

Our analyses, including both overall and subgroup assessments, found no significant differences between single-port and multi-port laparoscopic techniques in terms of operative time, conversion rates, and blood transfusion requirements, consistent with earlier reports. However, we did find a significant reduction in hospital stay length for single-port laparoscopy, likely due to the fewer incisions and reduced trauma. As for longer terms surgical outcomes, SPLH had same results compared with MPLH, showed the safety in SPLH. For long-term outcomes, the literature that reported on overall survival (OS) and progression-free survival (PFS) found no significant differences between the two surgical approaches. This suggests that single-port laparoscopy has similar safety and effectiveness compared to multi-port laparoscopy.

Regarding postoperative pain, available studies providing visual analog scale (VAS) pain scores showed no significant differences between the two surgical techniques [11, 24]. Some studies reported less postoperative pain in the single-port group compared to the multi-port group [23, 31], while Mereul et al. reported the opposite [32]. Larger, well-designed controlled trials are needed to confirm these findings. For long-term outcomes, the literature that reported on overall survival (OS) and progression-free survival (PFS) found no significant differences between the two surgical approaches. This suggests that single-port laparoscopy has similar safety and effectiveness compared to multi-port laparoscopy.

### Strength and limitations

To the best of our knowledge, this is the most comprehensive and up-to-date systematic review aimed at evaluating the efficacy, safety, and feasibility of single-incision laparoscopic surgery (SILS) compared to multi-port laparoscopic surgery (MPLS) in the treatment of endometrial cancer. Through a thorough analysis of perioperative outcomes and long-term surgical outcomes, it has been demonstrated that SILS is safe and effective in the treatment of endometrial cancer. The study results show that, when all surgical outcomes are comparable, SILS offers better postoperative recovery and less intraoperative bleeding. However, due to the limited number of included studies, most of which are retrospective, more randomized controlled trials (RCTs) are needed to further support our conclusions.

Our study has several limitations. First, there are only a few studies comparing single-port laparoscopy with multi-port laparoscopy for treating endometrial cancer, and only two are randomized controlled trials. To increase the sample size, we included high-quality prospective and retrospective studies. Second, although we applied strict GRADE criteria to manage the evidence and minimize selection bias, the inclusion of retrospective studies means the overall quality of our evidence is relatively low. Finally, despite including all high-quality studies comparing single-port and multi-port approaches, the small sample size means that, except for postoperative complications, the TSA analysis for other outcomes does not provide clear support for our conclusions. Therefore, more extensive and high-quality studies are needed to confirm our findings.

## Conclusion

We conducted a meta-analysis and systematic review based on a review of all current studies comparing single-port laparoscopy with multi-port laparoscopy for treating endometrial cancer. Our findings suggest that single-port laparoscopy provides short-term and long-term outcomes comparable in safety and effectiveness to multi-port laparoscopy. With its advantages in postoperative recovery and cosmetic results, single-port laparoscopy could become the preferred method for staging endometrial cancer surgeries.

## Supporting information

S1 Table Search strategy. (DOC)

S2 Table PRISMA 2020 checklist. (DOCX)

S1 Data (XLSX)

S2 Data (XLSX)
