# Supplementary material for: Surgical outcomes of single-port vs multi-port laparoscopic hysterectomy for endometrial cancer: A systematic review and meta-analysis
Source: PLoS One. 2024 Dec 9;19(12):e0314997. doi: 10.1371/journal.pone.0314997 (PMC11627385; doi:10.1371/journal.pone.0314997)
Supplement: S1 Table — (DOC) [file pone.0314997.s001.doc]

| #1 single incision |
| --- |
| #2 single site |
| #3 single port |
| #4 single access |
| #5 single trocar |
| #6 multi-port |
| #7 conventional |
| #8 standard |
| #9 traditional |
| #10 1or 2 or 3 or 4 or 5 or 6 or 7 or 8 or 9 (3,429,115) ^a^ |
| #11 laparoscopy |
| #12 laparoscopies |
| #13 11 or 12 (135,466) ^a^ |
| #14 10 and 13 (31,329) ^a^ |
| #15 laparo-endoscopic single site surgery |
| #16 14 or 15 (31,351) ^a^ |
| #17 Hysterectomy |
| #18 16 and 17 265(1,589) ^a^ |
| #19 Endometrial Neoplasm [Mesh] |
| #20 Endometrial Cancer |
| #21 Endometrial Carcinomas |
| #22 19 or 20 or 21 (45,882) ^a^ |
| #13 18 and 22 (224) ^a^ |
| Combined: 224 |

**S1 Table. Detailed search strategy.**

**PubMed search 2023.05.30**

All individual searches from each database inception date until 30 May 2023
a Number of articles combined for each search strategy.

**Embase search 2023.50.30**

| #1 Endometrial* AND adj3 |
| --- |
| #2 cancer* |
| #3 tumor* |
| #4 tumour* |
| #5 neoplas* |
| #6 carcinoma* |
| #7 malignan* |
| #8 Endometrial tumor/exp |
| #9 2 or 3 or 4 or 5 or 6 or 7 (7,111,613)**^a^** |
| #10 1 and 9 (70,399) **^a^** |
| #11 single incision or single port or single site or single access or single trocar |
| #12 laparoscopic |
| #13 hysterectomy |
| #14 12 and 13 and 14 (285)**^a^** |
| #15 11 and 15 (31) **^a^** |
| Combined: 30 |

**All individual searches from each database inception date until 30 May 2023**

**a Number of articles combined for each search strategy.**

**Cochrane search 2023.50.30**

| #1 Endometrial cancer |
| --- |
| #2 Endometrial* adj3 (tumor* OR tumour* OR neoplas* OR carcinoma* OR malignan* ) |
| #3 1 or 2 (2815) **^a^** |
| #4 laparoscopy |
| #5 single incision or single port or single site or single access or single trocar |
| #6 hysterectomy |
| #7 4 and 5 and 6 (184) **^a^** |
| #8 3 and 7 (10) **^a^** |
| Combined: 10 |

**All individual searches from each database inception date until 30 May 2023.**

**a Number of articles combined for each search strategy.**

**ClinicalTrials.gov search 2023.50.30**

| Condition/disease: Endometrial cancer or Endometrial Neoplasm |
| --- |
| Other terms: NA |
| Intervention/treatment: Single port laparoscopic surgery |
| Combined: 1 |

**All individual searches from each database inception date until 30 May 2023.**
